# Exploring the Impact of Dawn Phenomenon on Glucose-Guided Eating Thresholds in Individuals With Type 2 Diabetes Using Continuous Glucose Monitoring: Observational Study

**DOI:** 10.2196/46034

**Published:** 2023-08-11

**Authors:** Michelle R Jospe, Kari M Marano, Arianna R Bedoya, Nick L Behrens, Lacey Cigan, Vanessa Villegas, Michelle F Magee, David G Marrero, Kelli M Richardson, Yue Liao, Susan M Schembre

**Affiliations:** 1 Lombardi Comprehensive Cancer Center Georgetown University Washington, DC United States; 2 University of Arizona Tucson, AZ United States; 3 MedStar Health Diabetes and Research Institutes Washington, DC United States; 4 Department of Medicine Georgetown University School of Medicine Washington, DC United States; 5 University of Texas at Arlington Arlington, TX United States

**Keywords:** glucose-guided eating, dawn phenomenon, eating timing, eating behavior, type 2 diabetes, appetite regulation, blood glucose self-monitoring, food intake regulation, glycemic control, continuous glucose monitoring, glucose, appetite, diabetes, diabetic, type 2, monitoring, blood sugar, mobile phone

## Abstract

**Background:**

Glucose-guided eating (GGE) improves metabolic markers of chronic disease risk, including insulin resistance, in adults without diabetes. GGE is a timed eating paradigm that relies on experiencing feelings of hunger and having a preprandial glucose level below a personalized threshold computed from 2 consecutive morning fasting glucose levels. The dawn phenomenon (DP), which results in elevated morning preprandial glucose levels, could cause typically derived GGE thresholds to be unacceptable or ineffective among people with type 2 diabetes (T2DM).

**Objective:**

The aim of this study is to quantify the incidence and day-to-day variability in the magnitude of DP and examine its effect on morning preprandial glucose levels as a preliminary test of the feasibility of GGE in adults with T2DM.

**Methods:**

Study participants wore a single-blinded Dexcom G6 Pro continuous glucose monitoring (CGM) system for up to 10 days. First and last eating times and any overnight eating were reported using daily surveys over the study duration. DP was expressed as a dichotomous variable at the day level (DP day vs non-DP day) and as a continuous variable reflecting the percent of days DP was experienced on a valid day. A valid day was defined as having no reported overnight eating (between midnight and 6 AM). ∂ Glucose was computed as the difference in nocturnal glucose nadir (between midnight and 6 AM) to morning preprandial glucose levels. ∂ Glucose ≥20 mg/dL constituted a DP day. Using multilevel modeling, we examined the between- and within-person effects of DP on morning preprandial glucose and the effect of evening eating times on DP.

**Results:**

In total, 21 adults (59% female; 13/21, 62%) with non–insulin-treated T2DM wore a CGM for an average of 10.5 (SD 1.1) days. Twenty out of 21 participants (95%) experienced DP for at least 1 day, with an average of 51% of days (SD 27.2; range 0%-100%). The mean ∂ glucose was 23.7 (SD 13.2) mg/dL. People who experience DP more frequently had a morning preprandial glucose level that was 54.1 (95% CI 17.0-83.9; *P*<.001) mg/dL higher than those who experienced DP less frequently. For within-person effect, morning preprandial glucose levels were 12.1 (95% CI 6.3-17.8; *P*=.008) mg/dL higher on a DP day than on a non-DP day. The association between ∂ glucose and preprandial glucose levels was 0.50 (95% CI 0.37-0.60; *P*<.001). There was no effect of the last eating time on DP.

**Conclusions:**

DP was experienced by most study participants regardless of last eating times. The magnitude of the within-person effect of DP on morning preprandial glucose levels was meaningful in the context of GGE. Alternative approaches for determining acceptable and effective GGE thresholds for people with T2DM should be explored and evaluated.

## Introduction

Glucose-guided eating (GGE) is a timed eating paradigm that promotes metabolic homeostasis by deterring energy intake when circulating glucose is the primary source of fuel. GGE (historically called “hunger recognition” and “hunger training”) has been tested over the past 2 decades in adults without diabetes who often experience overweight or obesity [1-10]. GGE involves learning to eat only when physically hungry. People following GGE are trained to monitor perceived hunger and glucose levels and to associate symptoms experienced when glucose levels approximate (morning) fasting levels with being physically hungry. Eating according to GGE includes recognizing the symptoms of physical hunger and having preprandial glucose below a personalized threshold, which is computed as the average of 2 consecutive morning preprandial glucose levels [1,5,7,11]. Eating when glucose is below the GGE threshold requires postprandial glucose to return to a fasted state before initiating a subsequent eating event. Typically, the glucose monitoring stage of GGE (referred to as the training period) occurs over 2-4 weeks. Subsequently, trained individuals rely on their feelings of physical hunger and patterns of eating developed over the training period to guide meal timing without glucose monitoring. It is unknown whether the consequences of type 2 diabetes (T2DM), including the dawn phenomenon (DP; the increase in glucose levels between the nocturnal nadir and early morning [12]) impact the feasibility of GGE based on the personalized threshold.

GGE has been shown to be effective at promoting weight loss by limiting opportunities for energy intake. With high adherence (eg, completing an entry in a booklet for at least 60 out of the recommended 63 days), men lost 12.7 (95% CI 3.1-22.3) kg and women lost 5.2 (95% CI 3.1-7.4) kg after 6 months of GGE, which included 2 weeks of glucose and hunger monitoring [2]. Early research by Ciampolini et al [10] showed significant improvements in insulin sensitivity among 89 people without diabetes who followed GGE for 5 months. Similarly, we have shown that among women at risk for postmenopausal breast cancer and a BMI ≥27 kg/m^2^, those who followed a low-glucose eating pattern consistent with GGE over a 16-week intervention period have more favorable metabolic outcomes, including improvements in insulin resistance, than those who followed a high-glucose eating pattern, independent of weight changes [8]. Effects of GGE on insulin sensitivity are hypothesized to be indirectly linked through reduced adiposity and oxidative stress. This evidence suggests the potential of GGE as a behavioral treatment for T2DM.

Among people without evidence of T2DM, the threshold to guide decisions about meal timing is computed as the average of preprandial glucose for 2 consecutive mornings after fasting for at least 8 hours [1]. Using this method for computing and implementing GGE thresholds for people with T2DM is potentially complicated by the presence of the DP. DP has been associated with increased 24-hour mean glucose values, as well as increased glycemic variability [13-15]. Exaggerated morning preprandial glucose could result in an overly liberal glucose threshold that could promote eating without physical hunger while circulating glucose remains elevated, thereby impeding the onset of metabolic homeostasis. Yet, if the threshold is too strict, adherence to the protocol, particularly on mornings when glucose levels are exaggerated above the GGE threshold, may be unfeasible [1]. Both overly liberal and overly strict GGE thresholds could have a negative impact on the potential effectiveness of GGE among people with T2DM.

To examine the preliminary feasibility of using typically derived thresholds for implementing the GGE paradigm in people with T2DM, we examined the incidence and effect of DP on morning preprandial glucose measured by continuous glucose monitoring (CGM) in a convenience sample of adults with non–insulin-treated T2DM. Additionally, we examined the between- and within-person variability of morning preprandial glucose on days with and without DP and the impact of night eating as a predictor of DP.

## Methods

### Study Design

This was a completely remote observational study conducted from April to August 2021 that examined glucose patterns of people with T2DM using CGM. Study participants were recruited using ResearchMatch [16], a free and secure registry by the National Institutes of Health used to invite volunteers to take part in health research studies. Individuals who were registered on the ResearchMatch platform and potentially eligible for our study (eg, self-identified as having been diagnosed with T2DM and no reported use of insulin) were emailed a brief study description and invitation to contact the study team for additional study details. Individuals, who contacted the study team and remained interested in study participation, were screened for eligibility using a Research Electronic Data Capture (REDCap; Vanderbilt University) survey [17]. Inclusion criteria included a self-reported diagnosis of T2DM, being aged 18 years or older, having a self-reported weight and height equivalent to a BMI ≥30 kg/m^2^, reportedly having seen a primary care provider in the last 3 months, ability to speak and read English, and access to the internet and a smartphone, tablet, or computer for web-based meetings. The exclusion criteria were self-reported use of insulin (basal or fast-acting) and prior or current use of a CGM system. Those determined to be eligible were provided a digital informed consent document via REDCap to be signed remotely.

Once enrolled, participants were mailed a single, 10-day wear, disposable, Dexcom G6 Pro CGM and supplies (alcohol swabs, Skin Tac, and an overlay patch) and asked to complete a short demographic survey that included questions about medication use. Participants met with a trained study coordinator on a Health Insurance Portability and Accountability Act (HIPAA)–compliant Zoom (Zoom Video Communications Inc) health video chat session for assistance with the self-administration of their CGM sensors. Access to the participants’ CGM data via the Clarity app (Dexcom) was approved by all study participants. During the observational data collection period (up to 10 days), participants were blinded to their CGM data and asked to maintain their usual eating and activity patterns.

### Ethics Approval

The study was approved by the University of Arizona Human Subjects Protection Program Institutional Review Board (1904518491). Study participants provided informed consent prior to initiating the study. All study data were deidentified. All participants were provided with a US $50 Amazon gift card upon study completion.

### Measures

Participants were asked to report their first and last meal or snack times daily using REDCap surveys that were delivered daily by email or SMS text message (based on participant preference). Midway through the trial, participants were also asked to note the times of their lunch and dinner. For the remainder of this paper, “breakfast” refers to the first meal or snack; however, “dinner” and “last meal or snack” are not synonymous.

Morning preprandial glucose was defined as the CGM glucose level immediately prior to breakfast (between 6 AM and 10 AM). If breakfast occurred after 10 AM, the CGM glucose value at 10 AM was used.

The occurrence of DP was determined for valid days only. A valid day was one that had reported breakfast and last meal or snack, and no reported eating events between midnight and 6 AM. ∂ Glucose (mg/dL) was computed as the difference in nocturnal glucose nadir (between midnight and 6 AM) to prebreakfast glucose. If all nocturnal glucose values were above the prebreakfast glucose level, ∂ glucose was recorded as 0. ∂ Glucose greater or equal to the established threshold of 20 mg/dL was indicative of DP [13]. Rather than using a single valid day to determine the presence of DP, we used all obtained CGM data and defined DP at the day level (DP day or non-DP day) and as a continuous variable to reflect the percent of (valid) days that DP was experienced.

### Statistical Analysis

All descriptive variables were quantified at the person level. To analyze the effect of DP on morning preprandial glucose levels, we conducted multilevel analyses using within- and between-person means for DP with a random effect for participants. Participants wore CGM for an average of 10.5 (SD 1.1; range 6-11) days and provided an average of 8.1 valid days (SD 1.2; range 5-10), resulting in 170 valid days of data for analysis. Upon review of the collected data, 1 participant was excluded from the planned analysis due to overnight eating occurring each day of the observation period, which resulted in 0 valid days. This participant was removed from analyses using DP, resulting in an analytical sample of 21. Statistical analysis was performed using R (version 4.2.0; R Core Team).

## Results

Thirty-nine out of 85 screened individuals were eligible. The first 22 who agreed to participate consented and were enrolled, and 21 contributed to the analytical data set. Participant characteristics are described in Table 1.

Twenty of 21 participants (95%) experienced DP at least 1 day with an average of 51% (SD 27.2%; range 0%-100%) of days. Eleven of 21 participants (52.4%) experienced DP on at least 50% of the days. The mean ∂ glucose was 23.7 (SD 13.2) mg/dL.

**Figure 1:**
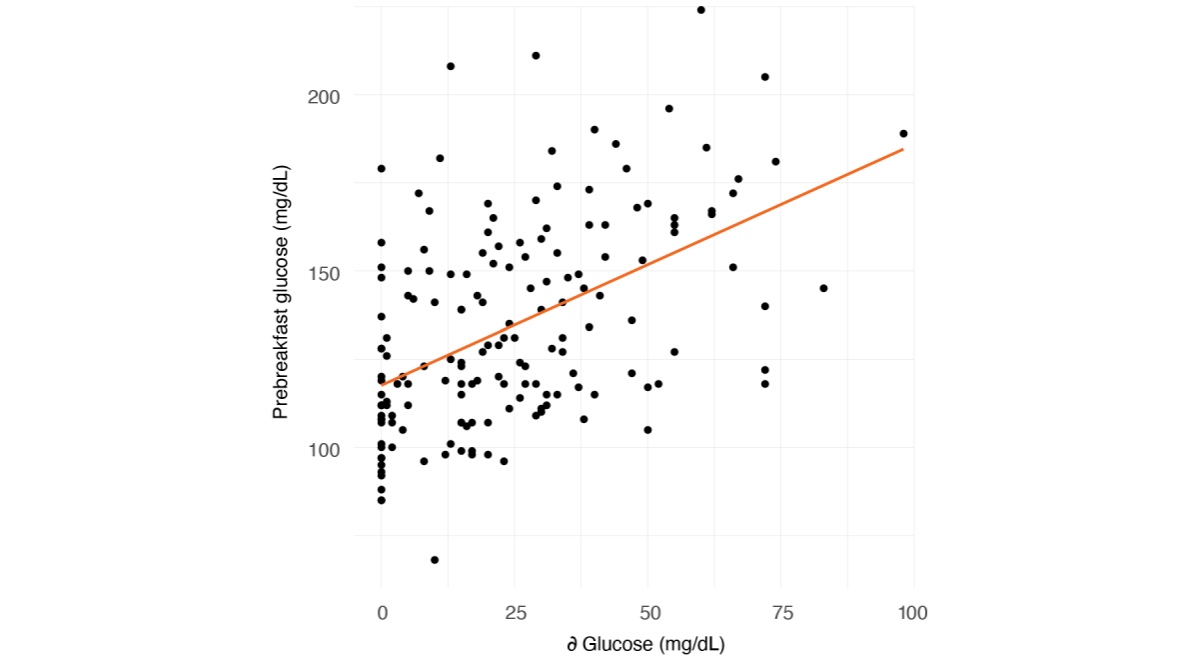
The correlation between the magnitude of the dawn phenomenon and prebreakfast glucose (*R*^2^=0.25, *P*<.001) in 21 subjects.

We observed an association between ∂ glucose and prebreakfast glucose of 0.50 (95% CI 0.37-0.60; *P*<.001; Figure 1). Table 2 summarizes the overall impact of DP on preprandial glucose levels at reported mealtimes. The timing of the last reported meal had no effect on prebreakfast glucose the next day (*P*=.97) or on the magnitude of DP (*P*=.85).

Multilevel models, which quantified the between- and within-person effects of DP on the morning preprandial glucose levels that would typically be used to compute GGE thresholds, showed that, on average, people who experience DP more frequently than the study sample average (between-person) had a prebreakfast glucose 54.1 (95% CI 17.0-83.9) mg/dL higher than those who experienced DP less frequently (*P*≤.001). Among within-person effects, prebreakfast glucose was 12.1 (95% CI 6.3-17.8) mg/dL higher on a DP day versus a non-DP day (*P*=.008). The complete multilevel regression analysis is been provided as Multimedia Appendix 1.

**Figure 2:**
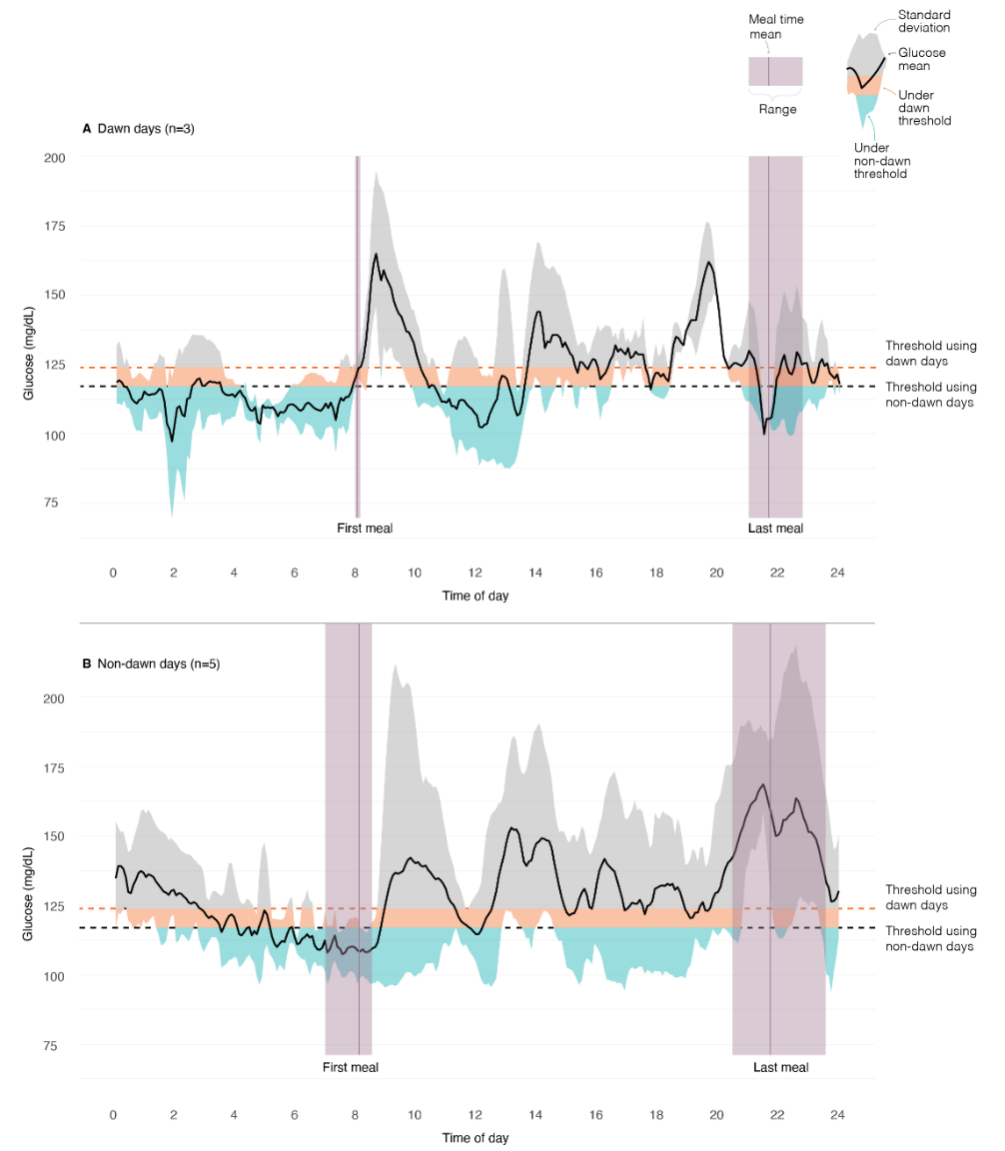
The diurnal glucose patterns on days (A) with and (B) without evidence of the dawn phenomenon from an exemplary study participant who had >75% valid days.

As a demonstration of the observed within-person effects of DP, Figure 2 shows the diurnal glucose patterns for DP and non-DP days from an exemplary study participant who had >75% valid days with a relatively equal balance of DP versus non-DP days. Applying thresholds derived from morning fasting glucose levels on 2 DP days (123.5 mg/dL) and 2 non-DP days (116.5 mg/dL), we provide an indication of the feasibility of GGE for the selected exemplary person. Figure 2 demonstrates how a GGE threshold derived from morning glucose levels on 2 DP days permits more opportunities to eat compared to a GGE threshold derived from morning glucose levels on 2 non-DP days. Specifically, for this exemplary participant, glucose levels drop to or below the DP day threshold 7 times and drop to or below the non-DP day threshold 4 times on DP days (Figure 2A) within their eating window. Similarly, on non-DP days (Figure 2B), glucose levels drop below the DP day threshold 8 times and non-DP day threshold only 2 times within their eating window.

**Table 1:** Participant characteristics (n=21).

Variable			Values
Age (years), mean (SD)			56.8 (11.7)
BMI (kg/m^2^), mean (SD)			39.2 (7.3)
**Sex, n (%)**			
	Female	13 (62)	
	Male	7 (33)	
	Nonbinary	1 (5)	
**Race, n (%)**			
	Black or African American	4 (19)	
	White	16 (76)	
	More than 1 race	1 (5)	
	Hispanic	2 (9)	

**Table 2:** Average CGM glucose at reported mealtimes on days with and without evidence of the dawn phenomenon (DP) (n=21).

Variable	All days	Non-DP days	DP days
Total observations, days	219	83	87
6 AM glucose (n=21), mg/dL	133.3 (21.8)	135.9 (27.8)	134.0 (21.2)
Breakfast time (n=21), h:mm	8:17 (1:04)	8:31 (1:05)	8:01 (1:05)
Prebreakfast glucose (n=21), mg/dL	132.6 (23.9)	125.2 (23.8)	138.6 (24.9)
Prelunch glucose (n=14^a^), mg/dL	128.4 (31.5)	129.2 (36.7)	132.6 (38.1)
Predinner glucose (n=15^a^), mg/dL	124.4 (23.8)	122.3 (27.0)	124.4 (27.3)
Last mealtime (n=21), hh:mm	20:33 (1:06)	20:42 (1:27)	20:31 (1:10)

^a^The request for prelunch and predinner glucose levels was added to the study protocol midstudy. Data are presented as mean (SD).

## Discussion

### Principal Results

This study examined the effect of DP on morning preprandial glucose in the context of implementing GGE in non–insulin-treated adults with T2DM. We found that DP was experienced on 51% of valid days and that, within-person morning fasting glucose levels were 12 mg/dL higher on DP days than on non-DP days. The effect was dose dependent, such that a greater magnitude of DP (∂ glucose) was associated with higher morning preprandial glucose levels. This magnitude of the effect would directly affect personalized GGE thresholds, as they are typically derived (from 2 morning fasting levels) and, subsequently, impact the potential feasibility or effectiveness of GGE among people with T2DM. Specifically, deriving a (higher) GGE threshold from 2 DP morning fasting glucose levels could cause GGE to be ineffective as it would permit frequent opportunities to eat. Alternatively, deriving a (lower) GGE threshold from 2 non-DP morning fasting glucose levels would render GGE unacceptable as it could overly restrict opportunities to eat. The results of this study indicate that alternative methods for deriving personalized GGE thresholds for people with T2DM should be explored and evaluated.

Research shows that DP is experienced by approximately 40%-50% of people with T2DM based on 1 to 2 days of glucose data [14,15,18]. Here, by categorizing days based on the DP definition, rather than participants, and including up to 11 days of CGM data per participant, we demonstrate that 52% (11/21) of participants experienced DP on ≥50% of the days of CGM wear. These findings suggest a high likelihood of deriving personalized GGE thresholds with at least 1 morning preprandial glucose level occurring on a DP day. The degree to which this might result in an overly liberal threshold would be person specific and directly related to the magnitude of DP on those days (eg, Figure 2). Evidence of this is further supported by the observed preprandial glucose levels at lunch and dinner times reported by our sample, which were, on average, lower than the morning preprandial glucose levels (Table 2). Obtaining sufficient intervention run-in data (ie, ≥5 days) on the presence of DP using CGM data would be beneficial for determining the need for an alternatively derived GGE threshold for adults with T2DM. Alternatively, based on the high prevalence of DP in this and other studies, one could assume that DP would be present on at least 1 of 2, consecutive days and develop an algorithm to account for the between-day variability, thereby eliminating the need for an extensive run-in period.

In the context of GGE, an optimal glucose threshold would promote glycemic control and weight loss (as needed), and improve insulin sensitivity [8,11]. The future implementation of GGE among people with T2DM will need to take DP into consideration when deriving personalized GGE thresholds. In our sample of people with T2DM, the average within-person magnitude of the DP effect was 12.1 (95% CI 6.3-17.8) mg/dL. This observation is consistent with a prior report by Monnier et al [13], who showed that the presence of DP is associated with a 12 mg/dL increase in 24-hour mean glucose. While this magnitude of effect might not have clinical implications related to the management of T2DM, we demonstrated here (Figure 2) that it likely has a marked impact on the number of opportunities to eat through the day. As such, alternative approaches to deriving GGE thresholds for people with T2DM could use a combination of 2 or more days of CGM run-in data and usual mealtime data to identify a more feasible threshold. It is also important to note, however, that the observed magnitude of effect is within that of adjustments made to GGE thresholds in previous studies among adults without diabetes. Specifically, approximately 20% of prior study participants of GGE interventions conducted by Schembre et al [7] benefited from adjustments to their assigned glucose thresholds. In these studies, adjustments made to GGE thresholds ranged from −5 mg/dL to +15 mg/dL. Threshold adjustments were made at the request of study participants who, early in the GGE training period, report concerns about adhering to the GGE paradigm. Specifically, for those who found their GGE threshold overly restrictive or who found their threshold overly permissive, following requested adjustments ultimately improved adherence or acceptability of the GGE paradigm. As such, an alternative method to deriving GGE thresholds for people with T2DM would be to use the typical, first 2 days of CGM data (only) and work directly with the study participant, early in the training period, to choose a feasible GGE threshold. From this and prior studies, it is unclear whether a single, alternative approach would produce desired adherence and efficacy results. Future research will be necessary.

### Strengths and Limitations

This study has several limitations. First, the sample size was relatively small, consisting of only 21 people. This may have affected the generalizability and precision of the observed estimates. However, the study is strengthened by the use of up to 11 days of CGM data to describe DP, rather than the standard 1-2 days, providing a greater number of observations. Second, the study relied on self-reported mealtimes and overnight eating, which may not have been entirely accurate or reliable. This could lead to errors in the calculation of morning preprandial glucose and DP. Future studies may consider using time-stamped food photographs to increase the accuracy of reported mealtimes. However, we are unaware of the collection of mealtimes and overnight eating being typical of DP research, which strengthened our ability to reliably identify DP. Third, while participants were instructed not to change their diet and activity, these behaviors were not monitored before or during the study. However, the study is strengthened by our choice to blind the data to reduce the likelihood of changes in response to glucose feedback. Finally, we did not examine whether perceived hunger differed preprandially for DP and non-DP days, which could be used as an indicator of threshold acceptability. We considered this as we were designing this study. GGE interventions typically ask participants to record preprandial hunger levels, and future interventions will do the same. However, the purpose of this observational study was to examine the usual diurnal pattern of glucose in T2DM, without participants following any intervention. As such, we did not want participants to change their dietary intake and, as research shows, recording perceived hunger ratings adds awareness to and modifies eating patterns.

### Conclusions

Among people with T2DM, the experience of DP was associated with elevated morning preprandial glucose levels of 12 mg/dL, which could result in an overly liberal GGE threshold. The feasibility of implementing GGE using thresholds computed from alternative approaches will be tested in future trials targeting people with T2DM. Determining how best to implement GGE for the millions of people with T2DM holds important public health implications as it has the potential to improve health and metabolic outcomes.

## Appendix

Multimedia Appendix 1 Multilevel model.

## Abbreviations

CGM: continuous glucose monitor
DP: dawn phenomenon
GGE: glucose-guided eating
HIPAA: Health Insurance Portability and Accountability Act
T2DM: type 2 diabetes

## Data Availability

The data sets generated and analyzed during this study are available from the corresponding author upon reasonable request.
