# Supplementary material for: Exploring the Impact of Dawn Phenomenon on Glucose-Guided Eating Thresholds in Individuals With Type 2 Diabetes Using Continuous Glucose Monitoring: Observational Study
Source: JMIR Form Res. 2023 Aug 11;7:e46034. doi: 10.2196/46034 (PMC10457696; doi:10.2196/46034)
Supplement: Multimedia Appendix 1 [file formative_v7i1e46034_app1.pdf]

Supplement 1: Multilevel model

Effect of dawn phenomenon on pre-breakfast glucose
Formula: breakfast glucose ~ between-person DP + within-person DP + (1 | ID)

Explanatory variables	Coefficient Estimate	Standard error	95% CI	P-value
Between-person DP	50.5	17.1	17.0, 83.9	0.008
Within-person DP	12.1	2.9	6.3, 17.8	<0.0001
Constant	133.3	4.5	124.4, 142.1	<0.0001

DP: dawn phenomenon
